# Anti-*Staphylococcus aureus* Activity of Volatile Phytochemicals and Their Combinations with Conventional Antibiotics Against Methicillin-Susceptible *S. aureus* (MSSA) and Methicillin-Resistant *S. aureus* (MRSA) Strains

**DOI:** 10.3390/antibiotics13111030

**Published:** 2024-10-31

**Authors:** Isidora Nikolic, Verica Aleksic Sabo, Damir Gavric, Petar Knezevic

**Affiliations:** Department of Biology and Ecology, Faculty of Sciences, University of Novi Sad, Trg Dositeja Obradovica 3, 21000 Novi Sad, Serbia; isidoran@dbe.uns.ac.rs (I.N.); verica.aleksic@dbe.uns.ac.rs (V.A.S.); damir.gavric@dbe.uns.ac.rs (D.G.)

**Keywords:** antibiotics, biofilm, carvacrol, *S. aureus*, synergism, thymol

## Abstract

Background: MSSA and MRSA strains are challenging human pathogens that can develop resistance to antibiotics, highlighting the need for alternative antimicrobial agents. Plant metabolites, particularly volatile phytochemicals, may offer promising antimicrobial properties. The aim was to evaluate the antimicrobial and antibiofilm efficacy of various commercial volatile phytochemicals from the terpene and terpenoid groups against reference MSSA and MRSA strains, focusing on synergistic effects in both binary combinations and combinations with antibiotics. Methods: The microdilution method was used to determine the minimum inhibitory concentrations (MICs) for antibiotics and phytochemicals. The checkerboard method assessed synergistic interactions between phytochemicals and between phytochemicals and antibiotics, while the time-kill method was used to confirm these results. Biofilm quantification was performed using the microtiter plate method to evaluate the effects of phytochemicals, antibiotics, and their binary combinations on the eradication of 48-h-old biofilms. Results: Carvacrol and thymol demonstrated the strongest anti-staphylococcal activity, while other terpene compounds showed weaker effects. In binary combinations, carvacrol and thymol exhibited synergy against one MSSA strain (FICI = 0.50) and with tetracycline and chloramphenicol (FICI = 0.28–0.50). Synergy was also noted with streptomycin sulfate against one MRSA strain (FICI = 0.31–0.50) and with other antibiotics, including gentamicin (FICI = 0.25–0.50) and oxacillin (FICI = 0.44). Additionally, effective combinations achieved over 50% biofilm removal at both minimum inhibitory and sub-inhibitory concentrations. Conclusions: Results showed that synergy varies based on strain sensitivity to chemical agents, highlighting their potential for personalized therapy. Despite the difficulty in removing preformed biofilms, the findings highlight the importance of combined treatments to enhance antibiotic effectiveness.

## 1. Introduction

*Staphylococcus aureus* is a Gram-positive coccoid bacterium commonly found on the skin and in the nasal mucosa of approximately 25–30% of asymptomatic individuals, as well as in other mucous membranes of humans and warm-blooded animals. These sites serve as a key reservoir for staphylococcal infections [1]. While it can cause mild skin infections and food poisoning, it is also responsible for serious nosocomial infections [2,3]. As a significant human pathogen, *S. aureus* has developed various resistance mechanisms and virulence factors, particularly through frequent antibiotic treatment. Notable resistance mechanisms include enzymatic inactivation (e.g., beta-lactamase), decreased affinity for target components (e.g., PBP2a protein in methicillin-resistant (MRSA) strains, d-Ala-d-Lac peptidoglycan precursor in vancomycin-resistant (VRSA) strains), alteration of cell membrane permeability for antibiotics, efflux pumps, and the production of biofilms [4,5]. The emergence of MRSA and VRSA strains presents a major challenge in the treatment of staphylococcal infections, particularly due to their ability to form biofilms [6].

The biofilm itself represents the aggregation of bacterial cells adhered to different biotic or abiotic surfaces and embedded in an extracellular matrix made of polysaccharides, proteins, extracellular DNA, and other molecules. Biofilms contribute to a wide range of infections and are a significant barrier to treatment because they protect the bacterial community from the immune system and the antibiotics themselves [7]. Resistance of biofilms occurs due to spontaneous mutation, which is not uncommon in staphylococci either [8]. Additionally, agents may be neutralized, and decreased permeability due to the biofilm matrix can facilitate the transfer of plasmids through conjugation and transformation, promoting the horizontal exchange of resistance genes [9]. Biofilms formed by staphylococci exhibit antibiotic resistance that is 1000 times greater than that of planktonic cells [10]. This makes biofilm an important factor in antimicrobial resistance and difficult-to-treat nosocomial infections.

In the absence of adequate chemotherapeutics, attention is drawn to new antimicrobial agents, including plant metabolites. Most studies have focused on determining the antibacterial activity of essential oils, which are a mixture of volatile, lipophilic, and aromatic substances originating from secondary plant metabolites [11]. The composition of essential oils is highly diverse and can vary significantly depending on factors such as the extraction method, plant age, genetics, environmental conditions, geographical climate, and harvest time [12,13]. Because of this variability, the antimicrobial activity of essential oils can also differ, making it challenging to identify which specific compounds are responsible for their effects. Most bioactive compounds in essential oils are terpenes and terpenoids, which show a diverse range of biological activities, including antimicrobial, anticancer, antiparasitic, neuroprotective, phytotoxic, anti-inflammatory, antioxidant, and antiallergic effects [14,15]. When these compounds are isolated as pure substances, it becomes easier to monitor and study their biological activities.

Carvacrol and thymol, the prominent and extensively studied terpenoids found in the essential oils of oregano and thyme [16], exhibit significant anti-staphylococcal activity [16,17,18,19,20,21,22,23]. These compounds inhibit bacterial growth by disrupting cell membrane integrity, leading to increased permeability and cell lysis [24,25]. They also interfere with bacterial metabolism by inhibiting key enzymes and modulating virulence-related gene expression [25,26]. Notably, carvacrol acts as an efflux pump inhibitor in both MSSA and MRSA strains, enhancing its efficacy against resistant strains [27,28,29]. Additionally, both compounds effectively inhibit biofilm formation and disrupt established biofilms [30,31,32,33,34,35], thereby improving treatment outcomes, as biofilms are critical for the persistence and the pathogenicity of *S. aureus*.

Given that bioactive compounds exhibit promising anti-staphylococcal effects while the efficacy of antibiotics declines, combining antibiotics with phytochemicals such as carvacrol and thymol offers a potential strategy to enhance their antimicrobial activity [22,27,36,37,38]. However, there is insufficient information about the synergistic effects between these compounds and certain antibiotics. In this study, we aimed to investigate the anti-staphylococcal activity of ten volatile phytochemicals to identify those suitable for combination with antibiotics. The time-dependent effects of these combinations on bacterial growth kinetics were analyzed to predict how synergistic combinations could be utilized in future clinical trials. Additionally, we examined the anti-biofilm activity of confirmed synergistic combinations of phytochemicals and antibiotics on planktonic cells and preformed staphylococcal biofilms, which are also of clinical importance.

## 2. Results

### 2.1. Anti-S. aureus Activity of Conventional Antibiotics and Volatile Phytochemicals Alone

The antibacterial activity of the selected volatile phytochemicals was detected for six out of ten tested strains (Table 1). Among the phytochemicals, carvacrol (CAR) had the best activity, with MIC values ranging from 128.0 to 203.2 μg mL^−1^ for MSSA and from 362.0 to 1024.0 μg mL^−1^ for MRSA. Thymol (THY) expressed slightly higher MICs compared to CAR for MSSA (256.0–724.01 μg mL^−1^) and for MRSA (512.0 ≥ 2048 μg mL^−1^). Compound (−)-terpinene-4-ol exhibited no activity against MRSA strains and weak activity against MSSA (1722.2–2048.0 μg mL^−1^). The terpene compounds, 3-carene and sabinene, showed activity against MRSA strains at higher concentrations (1024.0 ≥ 2048.0 μg mL^−1^ and ≥2048.0 ≥ 2048 μg mL^−1^, respectively). Also, 3-carene was active against one MSSA strain (1625.5 μg mL^−1^), while sabinene exhibited activity against three MSSA strains (2048.0 μg mL^−1^). Other compounds from terpene class—(R)-(+)-limonene, α-terpinene, γ-terpinene, and terpinolene—did not exhibit antimicrobial activity against any of the used *S. aureus* strains, even at the highest examined concentration (2048.0 μg mL^−1^), while only α-pinene expressed weak activity against MSSA strains (≥2048.0 μg mL^−1^) and one MRSA strain (2048.0 μg mL^−1^).

### 2.2. Binary Combinations of Phytochemicals and Combinations with Conventional Antibiotics

#### 2.2.1. Synergistic Combinations Determined by the Checkerboard Method

**Figure 1:**
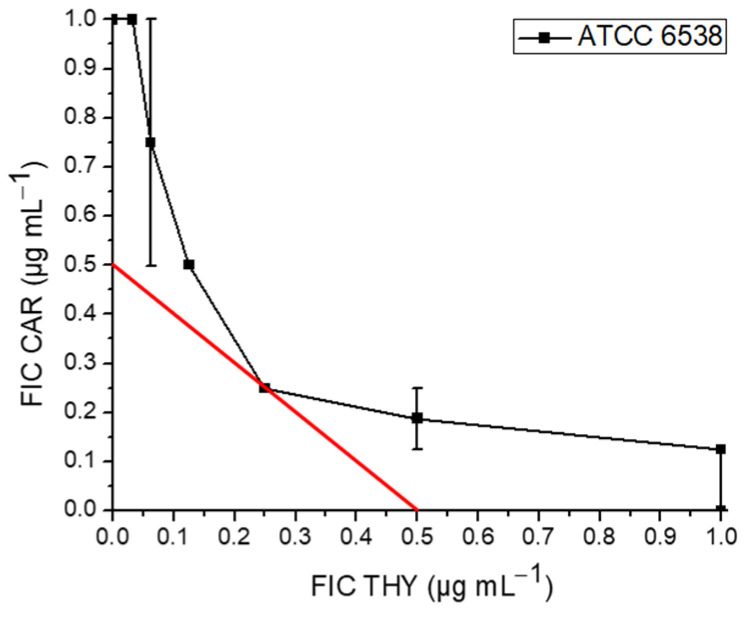
Effect of carvacrol (CAR) and thymol (THY) binary combination.

CAR and THY were used to characterize interactions between binary combinations or combinations with conventional antibiotics. Out of 28 examined binary combinations, 57.1% were characterized as synergistic (FICI ≤ 0.50). The combination of CAR and THY showed a synergistic effect against MSSA strain ATCC 6538 (FICI = 0.50) (Figure 1).

**Figure 2:**
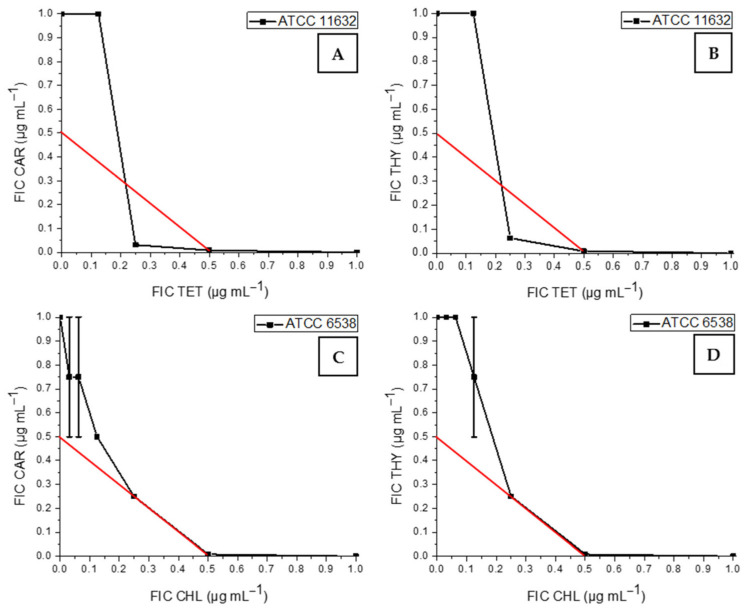
Synergistic effects of volatile phytochemicals and antibiotics against MSSA ATCC 11632 (**A**,**B**) and MSSA ATCC 6538 (**C**,**D**). CAR—carvacrol, THYthymol, TET—tetracycline, CHL—chloramphenicol.

A synergistic effect was also observed for CAR or THY in combination with TET against MSSA strain ATCC 11632 (FICI = 0.28 and FICI = 0.38, respectively) (Figure 2A,B). Similarly, CHL also showed a synergistic effect in combination with these phytochemicals against MSSA strain ATCC 6538 (FICI = 0.50; Figure 2C,D).

**Figure 3:**
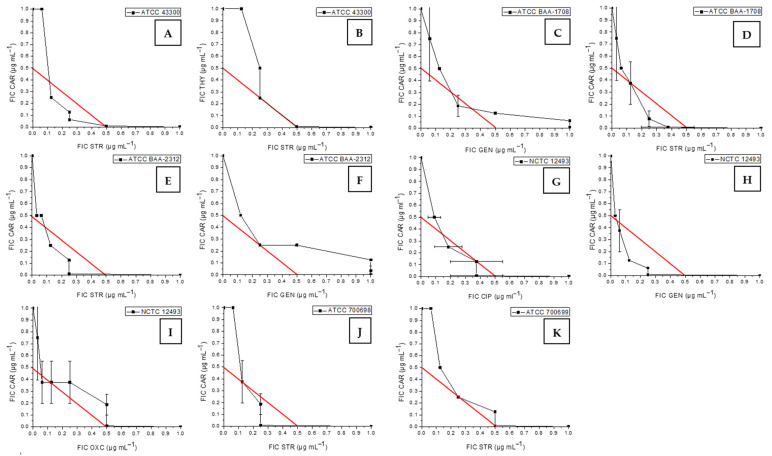
Synergistic effects of volatile phytochemicals and antibiotics against MRSA ATCC 43300 (**A**,**B**), ATCC BAA-1708 (**C**,**D**), ATCC BAA-2312 (**E**,**F**), ATCC 700699 (**K**), ATCC 700698 (**J**), and NCTC 12493 (**G–I**): CAR—carvacrol, THY−thymol, GEN—gentamicin, STR—streptomycin sulfate, CIP—ciprofloxacin, OXC—oxacillin.

Against MRSA strain ATCC 43300, synergy was observed only for the combination of CAR (FICI = 0.31; Figure 3A) or THY (FICI = 0.50; Figure 3B) with STR. Sinergy was also observed for MRSA strains ATCC 700699, ATCC 700698, ATCC BAA-1708, and ATCC BAA-2312 between CAR and STR (FICI = 0.50; FICI = 0.26, FICI = 0.32, and FICI = 0.26, respectively) (Figure 3D,E,J,K). Additionally, the combination of CAR and GEN showed synergy against MRSA strains ATCC BAA-1708, ATCC BAA-2312, and NCTC 12493 (FICI = 0.44, FICI = 0.50, and FICI = 0.25, respectively) (Figure 3C,F,H). Antibiotics CIP and OXC in combination with CAR also showed synergistic activity against MRSA strain NCTC 12493 (FICI = 0.38 and FICI = 0.44, respectively; Figure 3G,I).

#### 2.2.2. Growth Inhibition Kinetics of Synergistic Binary Combinations

In order to further characterize the obtained synergistic interactions, the kinetic growth inhibition of staphylococcal strains over a period of time was determined using the time-kill method. Out of 16 tested binary combinations characterized as synergistic by the checkerboard method, 9 were confirmed by the time-kill method (56.30%).

**Figure 4:**
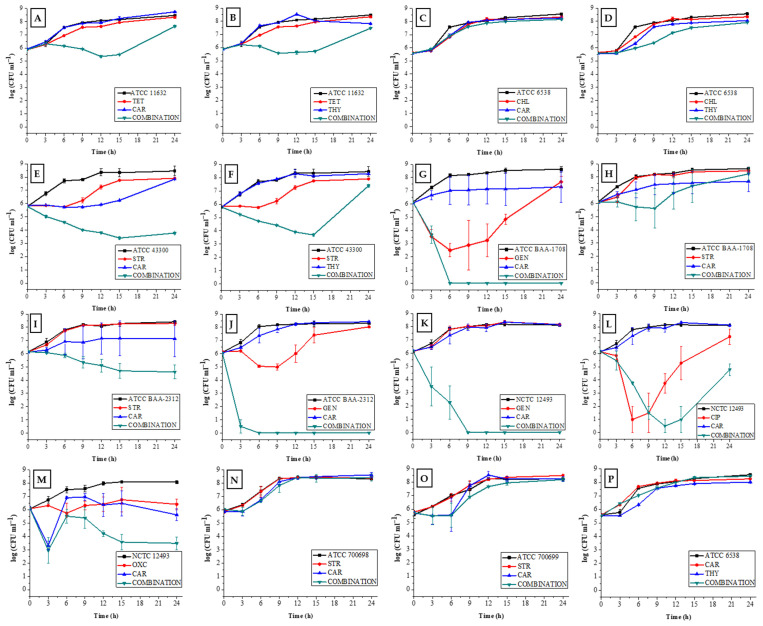
Time-kill assay for carvacrol and thymol in combination with antibiotics against MRSA ATCC 43300 (**E**,**F**), MRSA BAA-1708 (**G**,**H**), MRSA BAA-2312 (**I**,**J**), MRSA NCTC 12493 (**K**–**M**), MRSA ATCC 700698 (**N**), MRSA ATCC 700699 (**O**), MSSA 11632 (**A**,**B**), and MSSA ATCC 6538 (**C**,**D**), and in binary combination against MSSA ATCC 6538 (**P**). CAR—carvacrol, THY—thymol, TET—tetracycline, STR—streptomycin sulfate, CHL—chloramphenicol, GEN—gentamicin, CIP—ciprofloxacin, OXC—oxacillin.

For MSSA strain ATCC 11632, the CAR + TET combination maximally reduced the bacterial count by 0.50 log after 12 h compared to the initial count and by 0.85 log relative to the final count. Synergism was observed at 9, 12, and 15 h, with reductions of 1.70–2.40 log. However, after 24 h, there was no synergism (0.70 log reduction) (Figure 4A). The TYM + TET combination decreased the count by 0.30 log at 9 h and 1.00 log at 24 h, with similar transient synergisms (2.00–2.20 log) (Figure 4B). Although the combination of CAR or THY with CHL showed borderline synergism against MSSA (FICI = 0.50), the growth kinetics indicated a more additive to indifferent effect. After 24 h, bacterial counts increased by 2.60 log and 2.30 log for CAR + CHL and THY + CHL, respectively (Figure 4C,D).

For MRSA strain ATCC 43300, the CAR + STR combination reduced the bacterial count by 2.40 log after 15 h compared to the initial count, and by 4.70 log at 24 h compared to the final count. Synergism was observed from 12 to 24 h (2.10–4.10 log) (Figure 4E). The TYM + STR combination decreased the count by 2.00 log after 15 h, showing synergism at 12 and 15 h (3.20–3.90 log), but this was not maintained after 24 h (0.40 log reduction) (Figure 4F).

Significant synergism for combination CAR + GEN against MRSA strain ATCC BAA-1708 was observed, reducing the bacterial count by 2.50 log after 6 h and 7.30 log at 24 h (Figure 4G). In contrast, the CAR + STR combination did not show synergism against the same strain, with only a 0.50 log reduction after 9 h (Figure 4H).

For MRSA strain ATCC BAA-2312, the CAR + STR combination exhibited synergism, with reductions of 2.00–2.50 log from 12 to 24 h (Figure 4I). The CAR + GEN combination demonstrated a bacterial reduction of 6.10 log after 6 h, reaching 8.30 log after 24 h. This combination was most promising because synergism was detected after only 3 h of incubation, reducing the bacterial count by 6.30 log (Figure 4J).

The CAR + GEN combination on MRSA strain NCTC 12493 reduced bacteria by 2.60 log after 3 h and became undetectable after 9 h, exceeding 8.20 log by the end of incubation (Figure 4K). Against the same strain, the combination of CAR + CIP had the greatest reduction after 12 h of 5.60 log compared to the initial count. The synergism started after 12 h, with a 3.20 log reduction and 2.51 log after 24 h (Figure 4L). Similar to GEN + CAR, the OXC + CAR combination decreased the bacterial number by 3.10 log after 3 h compared to the initial number and the synergism started at 12 h, with a consistent reduction of 2.14 log after 24 h (Figure 4M).

No synergism was confirmed for CAR + STR against MRSA strains ATCC 700698 and ATCC 700699 (Figure 4N,O) or for CAR + THY against MSSA strain ATCC 6538 (Figure 4P). Increases in bacterial counts were noted for ATCC 700698 (0.10 log) and minimal reductions were noted for ATCC 700699 (0.05 log) and ATCC 6538 (0.11 log) after 24 h.

### 2.3. Biofilm Quantification

To evaluate the effects of antibiotics and essential oil compounds, both individually and in combination, it was necessary to examine the ability of biofilm formation. Based on the obtained results, the majority of strains produced biofilm better after 48 h, and the production was stronger in the presence of glucose (Table 2). Strains that expressed moderate to strong biofilm-forming ability under all experimental conditions were MSSA strain ATCC 6538 and MRSA strain ATCC BAA-2312, while MSSA strain ATCC 11632 did not form biofilm during the experiment.

#### 2.3.1. Biofilm Eradication Activity of Antibiotics and Phytochemicals Alone

Strains MSSA ATCC 6538 and MRSA ATCC BAA-2312, characterized as strong biofilm producers, were selected for the experiment to emphasize the effectiveness of the compound on established biofilms. The biofilm eradication ability of the antibiotics and phytochemicals against MSSA strain ATCC 6538 was good when CHL or CAR was applied at MICs, removing 51.6 and 56.5% of the biofilm, respectively. Other antibiotics and THY were active against the same strain at super MICs (Table 3). The best planktonic cell removal activity was achieved by CAR at an MIC of 89.8%, so the PEC value was the same as the MIC value of CAR. Except for CAR, THY at 2 × MIC (PEC) removed 54.6% of planktonic cells, whereas CHL and other antibiotics showed no activity against planktonic cells.

For MRSA strain ATCC BAA-2312 (Table 3), the best biofilm eradication activity was achieved using CTX, CHL, TET, and OXC (87.7%, 94.0%, 92.2%, and 91.0%, respectively) at the MIC, with PEC values matching the MICs (82.2, 65.0, 54.0, and 71.1%, respectively). Similar to the MSSA strain, other antibiotics required higher concentrations to remove the biofilm and planktonic cells of MRSA strain ATCC BAA-2312, as well as CAR.

#### 2.3.2. Biofilm Eradication Activity of Antibiotics, and Phytochemicals in Combination

For the current study, binary combinations were tested based on the synergistic effects observed using the checkerboard method.

All three combinations—CHL + THY, CHL + CAR, and CAR + THY—expressed synergism because 1/4 × MIC combinations of both agents were sufficient for biofilm removal of MSSA ATCC 6538 (78.3, 79.3, and 74.6%, respectively). Also, the combination of CHL + THY led to a decrease in the concentrations required for the removal of planktonic cells (1/4 × MIC for both agents), while CAR in combination with CHL or THY led to a decrease in their PEC values (1/4 × MIC). The biofilm of MRSA strain ATCC BAA-2312 was more resistant to combinations of CAR with STR or GEN (Table 4). This study hypothesizes that CAR influences the ability of antibiotics to eradicate biofilms and remove planktonic cells at MICs compared to using antibiotics alone at their MICs. However, no synergistic effect was observed. Unlike the MSSA strain, increasing the concentrations of the combinations simultaneously increased the percentage of biofilm eradication and removal of planktonic cells of the MRSA strain, indicating the necessity of applying MICs and super MICs for these binary combinations.

## 3. Discussion

### 3.1. Anti-S. aureus Activity of Volatile Phytochemicals

Staphylococcal antibiotic resistance is becoming an increasingly significant concern for human health, highlighting the need to explore alternative antimicrobial agents. Phytochemicals, natural compounds derived from plants, have emerged as promising solutions due to their diverse biological activities.

This study investigates the antimicrobial effects of specific phytochemicals, among which CAR and THY have shown significant activity against various MSSA and MRSA strains. Previous studies have reported comparable MICs for CAR against various strains, including ATCC 25923, ATCC 6538, and certain MRSA strains [17,18,30,39,40,41]. However, varying MIC values have been observed for MRSA strains ATCC 43300, ATCC 700698, and ATCC 33591 [17,19,42], as well as for MSSA strain ATCC 6538 [43].

For THY, similar results have been noted against ATCC MSSA and MRSA strains [17,30,39,40,43], although some studies indicate lower MICs for MRSA strains ATCC 33591, ATCC 43300, and ATCC 700698 [19,41,42]. These discrepancies may arise from variations in the methods used for MIC determination.

Data on other reference strains examined in this study are limited in the literature. Terpinene-4-ol exhibited weaker activity compared to CAR and THY, consistent with findings from studies involving strains ATCC 25923, ATCC 6538, and ATCC 43300 [44,45,46,47,48]. Although terpinene-4-ol exhibits antimicrobial activity against *S. aureus* through mechanisms such as membrane disruption and enzymatic inhibition, its lower effectiveness compared to CAR or THY may be attributed to differences in chemical structure, hydrophobicity, stability, and mechanisms of action [46,49].

Higher concentrations of terpene compounds, such as 3-carene and sabinene, were required to inhibit MRSA strains, as confirmed for MRSA ATCC 43300 [42]. Also, the weak activity of other terpene compounds like (R)-(+)-limonene, α-terpinene, γ-terpinene, and terpinolene has been reported for ATCC MSSA and MRSA strains [39,40,42,43,44,47,50,51,52,53,54].

Based on the results obtained and the available literature, only CAR and THY, among the examined phytochemicals, demonstrate significant anti-staphylococcal activity. This may be attributed to the presence of functional groups in terpenoids, which generally enhance their antimicrobial efficacy against staphylococci compared to terpenes.

Additionally, CAR was effective against both MSSA and MRSA strains, indicating its potential as a single-agent treatment for staphylococcal infections. In contrast, THY’s effectiveness was more limited, which may restrict its use for MRSA infections. While higher concentrations of both agents might be necessary, the potential toxicity of these concentrations should be tested. Considering that antibiotics can cause various side effects, combining them with these phytochemicals could provide mutual benefits by reducing the required concentrations of both agents and decreasing the emergence of resistance in bacteria.

### 3.2. Anti-S. aureus Activity of Antibiotics and Phytochemicals in Combination

#### 3.2.1. Anti-*S. aureus* Activity of Combinations

Due to emerging staphylococcal resistance, combined therapy presents an effective strategy for enhancing antibiotic efficacy. This approach involves using antibiotics in combination with other agents, such as phytochemicals. Additionally, phytochemicals can also be combined to explore potential synergistic effects. Previous studies have reported a predominant additive effect for the combination of CAR and THY against various *S. aureus* strains [16,17,20,55]. However, some studies noted indifferent or even antagonistic effects against different strains [21,56]. These variations highlight that CAR–THY interactions depend on multiple factors, emphasizing the need for further characterization.

In contrast to our findings, Miladi et al. reported a two-fold decrease in TET concentration when combined with CAR or THY against MSSA strain 25923 [27], which we did not observe. Similar reductions in TET concentrations with these phytochemicals were noted for other *S. aureus* strains [28,36]. Discrepancies may be attributed to differences in methods, concentrations used in binary combinations, strain sensitivities, and unspecified methodological variations. For instance, borderline synergism was observed for CAR or THY combined with TET on MSSA strain ATCC 29213 (FICI = 0.53) and MRSA strain ATCC 43300 (FICI = 0.38) [57], contrasting with our results. Similar inconsistencies are noted with combinations of CAR or THY with GEN, where synergism was reported for MSSA ATCC 29213 (FICI = 0.25 and FICI = 0.19, respectively) and MRSA ATCC 43300 (FICI = 0.19 and FICI = 0.25, respectively).

Previous studies demonstrated that THY + GEN reduced the MIC of GEN by 16 times on ATCC 12624 [37], while the same combination’s FICI was 0.38 on ATCC 9144 [22]. Our study uniquely identified significant effects of CAR + GEN and THY + GEN combinations against *S. aureus* strains. Additionally, we highlight substantial synergistic activity of CAR or THY with CIP, resulting in an eight-fold reduction in the MIC of CIP. This aligns with previous findings and supports reported additive interactions for THY + CIP combinations against MSSA ATCC 25923 and two MRSA strains [38,58].

Additionally, our results support the synergistic effect of CAR’s combination with CIP against *Bacillus cereus* ATCC 11778 and *Escherichia coli* ATCC 11775 [59], while THY and VAN exhibited additive effects against MRSA ATCC BAA-1717 [31]. However, CAR or THY showed indifferent or antagonistic effects with methicillin and penicillin G against MRSA ATCC 43300 [60]. Since these antibiotics target cell wall synthesis, similarly to CAR and THY, synergism may not occur when they target the same sites in certain *S. aureus* strains.

To the best of our knowledge, no data exist on the effects of CAR combined with STR, or CAR or THY combined with CHL and OXC on *S. aureus* strains. Our results indicate a significant effect of these combinations. It is crucial to note that the diversity of results among researchers can be attributed to the lack of standardized methods for testing binary combinations. Adhering to established principles is essential for the reproducibility of experiments and the effective clinical application of phytochemicals.

#### 3.2.2. Time Kinetics of Bacterial Growth Inhibition for Synergistic Interaction

While checkerboard assays provide a quick preliminary assessment, time-kill assays offer a more accurate representation of an antibiotic’s effectiveness over time and help identify the time point at which synergism occurs. For instance, Aleksic Sabo et al. confirmed synergy between CAR or THY and CIP against multidrug-resistant *Acinetobacter baumannii* strains [61], highlighting the potential of these combinations against drug-resistant bacteria, while Gan et al. observed synergy for THY + STR and THY + GEN combinations against *S. aureus* ATCC 9144 [22].

Our time-kill assay results indicate that the most effective antibiotics in combination with CAR or THY against staphylococci were GEN, STR, and TET, while CIP and OXC exhibited slightly weaker activity. Notably, GEN, STR, and TET target the 30S subunit of the ribosome. The enhanced activity of these antibiotics, when combined with terpenoids such as CAR and THY, which act on the bacterial cell membrane, suggests a potential mechanism where terpenoids facilitate the antibiotics’ entry into the cytoplasm.

Future research should focus on elucidating the mechanisms underlying these synergistic interactions and evaluating the cytotoxicity of these combinations to ensure their safe application in clinical settings. Given that CAR and THY are food additives with low toxicity and are listed as Generally Recognized As Safe (GRAS) by the U.S. Food and Drug Administration (US FDA), they hold promise for both food and medical applications, provided safe dosages are established [62,63,64,65].

For effective therapy that combines antibiotics and phytochemicals, it is essential to consider resistance to both types of agents. Investigating potential cross-resistance is crucial, as understanding how *S. aureus* strains respond to each can inform treatment strategies, mitigate resistance risks, and enhance the effectiveness of combination therapy.

### 3.3. Biofilm Formation

The choice of growth medium is crucial for studying biofilm formation. While MHB is commonly used, other media such as Brain Heart Infusion Broth (BHIB) and Tryptic Soy Broth (TSB) can also be effective. The addition of glucose, particularly at a concentration of 1% in TSB, can promote and stabilize biofilm formation in MSSA and MRSA strains [66]. This may explain why the strains tested in our study formed better biofilms in the presence of glucose in MHB.

However, enhanced biofilm formation due to glucose poses significant risks in hospital settings, particularly for patients with diabetes. Waldrop et al. demonstrated that clinical isolates have been shown to develop strong biofilms at glucose concentrations commonly encountered in clinical practice [67]. It is speculated that biofilm growth may be induced by the lower pH resulting from glucose utilization by bacterial cells [67]. This situation can be particularly dangerous due to the reduced efficacy of antibiotics against these stronger biofilms, leading to recurrent infections, as biofilms are a source of infection and contribute to the spread of antibiotic resistance.

These findings highlight the diverse phenotypic and genotypic characteristics of each strain and species under varying conditions, which can affect biofilm development. This is consistent with Croes et al., who demonstrated that staphylococcal biofilm formation depends on clonal lineage and genetic background [68].

To account for these variations, different assays for biofilm quantification are necessary [16,18,19,20,21,22,27,28,31,36,37,38,41,42,43,44,45,46,47,48,49,50,51,52,53,54,55,56,57,58,59,60,61,62,63,64,65,69]. It is important to note that each step in the microtiter method is crucial for the success of the experiment [70]. Key factors include the substrate used for inoculation (along with the size of the bacterial inoculum) and the number of washes between biofilm formation, fixation, staining, and decolorization. The rinsing technique employed also plays a significant role.

While this method, like other in vitro approaches, may not accurately reflect biofilm formation under in vivo conditions, it can be instrumental in identifying new anti-biofilm solutions through the combined effects of different agents.

#### 3.3.1. Biofilm Eradication Effects of Individual Agents

Staphylococcal biofilms present significant challenges in medicine, veterinary practices, and various industries, highlighting the urgent need to identify compounds capable of disrupting established biofilms resistant to antibiotics and disinfectants. The removal of mature biofilms is more challenging than inhibiting early-stage formation [18,30,32,55,71]. Using super MICs of CAR against MRSA strains and THY against MSSA strains resulted in higher biofilm removal rates. However, higher CAR concentrations decreased biofilm removal and increased planktonic cell removal of MSSA strains. This could be a defense mechanism that promotes biofilm formation to protect planktonic cells, which were barely present in the medium. Additionally, while higher concentrations of STR removed the biofilm, they decreased planktonic cell removal, suggesting that the biofilm was detached while the cells were in the stationary phase, during which they were sufficiently resistant to the action of a super MIC of STR.

#### 3.3.2. Biofilm Eradication Effects of Combinations

It is important to note that the sub-inhibitory concentrations, in almost all combinations of CAR with THY and antibiotics (1/2 and 1/4 × MIC), did not stimulate biofilm production, contrasting with typical effects seen with essential oils [72]. The CAR + THY combination showed potential against both monospecies and mixed biofilms [33], and according to our results on *S. aureus* monospecies, biofilms at super MICs. The choice and concentration of antibiotics are critical in addressing persisters within biofilms [73]. Certain antibiotics like CIP and GEN allow survival at sub-inhibitory levels, while others like VAN and OXC may enhance biofilm production [74].

A combination of antibiotics and phytochemicals may provide a synergistic approach to mitigate survivor cells, although many studies have yet to investigate the underlying mechanisms of this synergy. It is suggested that synergistic effects often arise from multi-target actions, where antibiotics primarily inhibit DNA or protein synthesis, while phytochemicals disrupt cell membranes [75]. Due to its hydrophobic nature, CAR interacts with the lipid bilayer, causing leakage of cellular components [23,76] and THY damages membranes by altering NADPH levels and increasing lipid peroxidation [24,77]. However, the mechanisms are likely more complex within biofilms. Our findings suggest that future strategies to combat biofilm formation may involve the use of synergistic combinations of natural products with antibiofilm properties. This approach could potentially provide more effective and targeted treatments for biofilm-related infections. However, researchers face significant challenges due to experimental variability and strain differences, underscoring the need for standardized terminology and methodologies in biofilm research [34,35,71,78].

## 4. Materials and Methods

### 4.1. Materials

#### 4.1.1. Bacterial Strains and Culture Conditions

Three reference strains of MSSA (ATCC 11632, ATCC 25923, and ATCC 6538) and seven reference strains of MRSA (ATCC 43300, ATCC 33591, ATCC BAA-1708, ATCC BAA-2312, ATCC 700699, ATCC 700698, and NCTC 12493) from the American Type Culture Collection (Rockville, MD, USA) were used for the antimicrobial tests. Bacterial strains were stored in Luria Bertani broth (LBB) (HiMedia, Mumbai, India) with glycerol supplementation (10% *v*/*v*) at −70 °C.

#### 4.1.2. Antimicrobial Agents

In total, ten commercially available volatile phytochemicals were examined: α-pinene, (R)-(+)-limonene, sabinene, 3-carene, α-teprinene, γ-terpinene, terpinolene, (-)-terpinene-4-ol, thymol, and carvacrol (Sigma-Aldrich, Steinheim, Germany), as well as ten conventional antibiotics: ceftazidime (CAZ) (PharmaSwiss, Beograd, Serbia), ceftriaxone (CTX) (Galenika, Zemun, Serbia), streptomycin sulfate (STR) (Sigma-Aldrich, Germany), chloramphenicol (CHL) (Sigma-Aldrich, Germany), tetracycline (TET) (Sigma-Aldrich, Germany), amoxicillin clavulanic acid (AMC) (Medochemie, Limassol, Cyprus), gentamicin (GEN) (Galenika, Serbia), ciprofloxacin (CIP) (Acros Organics, Geel, Belgium), oxacillin (OXC) (Sigma-Aldrich, Germany), and vancomycin (VAN) (Liophilchem, Roseto degli Abruzzi, Italy).

### 4.2. Anti-S. aureus Activity of Conventional Antibiotics and Volatile Phytochemicals Alone

#### Microdilution Method

In order to determine minimal inhibitory concentrations (MICs) of the tested antibiotics and phytochemicals, the microdilution susceptibility testing method was used [79], slightly modified for both types of compounds. Overnight cultures were grown on Müller Hinton agar (MHA) for 24 h at 37 °C before MIC determination. Müller Hinton broth (MHB) was inoculated with the bacteria to obtain a final concentration of 2 × 10^6^ CFU mL^−1^.

Stock solutions of the tested antibiotics were prepared in sterile distilled water, while phytochemicals were dissolved in 100% dimethyl sulfoxide (DMSO), ensuring a final DMSO concentration of ≤0.8%. Phytochemicals were tested in two-fold serial dilutions ranging from 2048.0 to 16.0 μg mL^−1^, and antibiotics were diluted from 64.0 to 0.5 μg mL^−1^. After the addition of equal volumes of bacterial inoculum (1:1, *v*/*v*) into each well, the final bacterial count was approximately 1 × 10^6^ CFU mL^−1^. The microtiter plates were tightly sealed with parafilm and incubated for 24 h at 37 °C. MIC was visually determined as the lowest concentration of agent that inhibited bacterial growth, with final confirmation using triphenyltetrazolium chloride (TTC) (0.1% *w*/*v*) and optical density (OD) measurement at 540 nm.

Phytochemical concentrations were recalculated from μL mL^−1^ or percentage to μg mL^−1^ based on their densities (Table 1) to ensure comparability [80].

### 4.3. Anti-S. aureus Activity of Binary Combinations

#### 4.3.1. Pre-Checkerboard Method and Checkerboard Method

The checkerboard method was used to determine interactions between antibiotics and phytochemicals, and binary combinations between different phytochemicals [80]. Before checkerboard testing, the potential synergistic interactions were examined by combining 1/4 × MIC of both agents (pre-checkerboard method). If bacterial growth was not observed for a combination, the checkerboard method was applied (Supplementary Materials Table S1).

Only antibiotics with MICs ranging from 1.0 to 64.0 μg mL^−1^ and phytochemicals with MICs from 128.0 to 1024.0 μg mL^−1^ were used in the pre-checkerboard and checkerboard methods. These ranges were selected to include concentrations that can be used in vivo and for clinical purposes. Antibiotics and phytochemicals were two-fold serially diluted in the checkerboard method, ranging from 1 × MIC to 1/32 × MIC and 1 × MIC to 1/128 × MIC, respectively.

The Fractional Inhibitory Concentration Index (FICI) [81] was calculated to assess the interaction type between agents:

$$\mathrm{FICI}=\frac{\mathrm{MIC}\;\mathrm{of}\;\mathrm{component}\;A\;\mathrm{in}\;\mathrm{mixture}}{\mathrm{MIC}\;\mathrm{of}\;\mathrm{component}\;A}+\frac{\mathrm{MIC}\;\mathrm{of}\;\mathrm{component}\;B\;\mathrm{in}\;\mathrm{mixture}}{\mathrm{MIC}\;\mathrm{of}\;\mathrm{component}\;B}$$

#### 4.3.2. Time-Kill Curve Method

The kinetics of bacterial growth inhibition for synergistic interaction, over a period of time, between volatile phytochemicals and antibiotics were performed using a slightly modified time-kill method [80,82]. Modifications were made during the setup of the experiment, which was conducted in parallel across four test tubes. The specific modifications were as follows: (I) bacterial count was 5 × 10^5^ CFU mL^−1^: MSSA strains (ATCC 11632, ATCC 6538) and MRSA strains (ATCC 43300, ATCC BAA-1708, ATCC BAA-2312, ATCC 700699, ATCC 700698, NCTC 12493); (II) sub-inhibitory antibiotic concentration and bacteria (1/4 × MIC): MSSA strains with TET or CHL and MRSA strains with STR, GEN, CIP, or OXC; (III) sub-inhibitory concentration of volatile phytochemical and antibiotics (1/4 × MIC): MSSA and MRSA strains with CAR or THY; and (IV) sub-inhibitory concentrations of antibiotics and volatile phytochemicals with bacteria (1/4 × MICs for both agents). After incubation periods of 0, 3, 6, 9, 12, 15, and 24 h at 37 °C, the bacterial counts (CFU mL^−1^) were determined on MHA by the spreading plate method. A reduction in bacterial count of ≥2 log compared to a more effective agent administered alone was considered a synergistic interaction [83].

### 4.4. Biofilm Quantification Assay

The microtiter plate method described by [84] was used for biofilm quantification of three MSSA and seven MRSA strains. The test was performed using 24-h-old cultures. To compare biofilm production, bacterial suspensions were adjusted to a cell density of 2 × 10^6^ CFU mL^−1^ in MHB and MHB supplemented with 1% glucose. Microtiter plates were incubated for 24 h and 48 h at 37 °C, and optical density (OD) was measured at 570 nm using a MultiscanGo microtiter plate reader (Thermo Scientific, Vantaa, Finland).

The biofilm production intensity was classified into four categories according to the cut-off of ODc values for negative controls: (I) nonadherent: OD ≤ ODc; (II) weakly adherent: ODc ≤ OD ≤ 2 × ODc; (III) moderately adherent: 2 × ODc ≤ OD ≤ 4 × ODc; (IV) strongly adherent: 4 × ODc ≤ OD.

#### 4.4.1. Single-Agent Biofilm Eradication Assay

The effect of phytochemicals and antibiotics alone on the eradication of 48-h-old biofilm was examined using a modified microtiter plate method [84]. Two strains, MSSA ATCC 6538 and MRSA ATCC BAA-2312, were grown on MHB supplemented with 1% glucose for 48 h at 37 °C.

Only antibiotics with MICs ranging from 1.0 to 64.0 μg mL^−1^ and phytochemicals with MICs ranging from 128.0 to 1024.0 μg mL^−1^ were used. Based on this, for MSSA biofilm eradication, four antibiotics (CHL, CAZ, VAN, STR) and two phytochemicals (CAR, THY) were tested; for MRSA biofilm eradication, six antibiotics (CHL, VAN, STR, CTX, TET, GEN, OXC) and one phytochemical (CAR) was tested.

Prepared concentrations of agents for single treatments ranged from 1 × MIC to 4 × MIC and were added (200 µL per well) after the biofilm was washed and the plates dried. The microtiter plates were then incubated at 37 °C for 24 h. The planktonic cell eradication (PEC) assay was performed first by transferring 100 µL of the well content to a new microtiter plate, followed by the addition of 90 µL of double-strengthened MHB supplemented with 1% glucose and 10 µL of 1% TTC. The plates were incubated at 37 °C for 3 h, and absorbance was read spectrophotometrically at 540 nm. PEC (planktonic eradication concentration) was defined as the minimum concentration that prevented the appearance of red color from TTC, indicating inhibition of planktonic cell release.

Biofilm eradication concentration (BEC) was quantified using the method described in Section 4.4. BEC was determined after the PEC assay and expressed as a percentage, with the percentage of biofilm and planktonic cell removal calculated based on the mean OD values for treatments in relation to the control. Obtained negative OD values were considered as zero.

#### 4.4.2. Binary Combination Biofilm Eradication Assay

The principle of the experiment remains the same as described in Section 4.3.2, where the eradication of biofilms was investigated using single agents. For the MSSA strain, three binary combinations (CAR + THY, CAR + CHL, and THY + CHL) were selected, while for the MRSA strain, two binary combinations (CAR + GEN and CAR + STR) were examined. For the synergistic combinations, the concentration range was from 1/4 × MIC to 4 × MIC.

### 4.5. Data Analysis

Each experiment was performed in triplicate and independently repeated at least twice. MIC values were calculated as geometric means (GMs) to reduce the impact of extreme outliers.

$$GM=\sqrt[{n}]{x_{1}\times x_{2}\times \ldots \times x_{n}}$$

Other results were averaged and expressed as means ± standard errors. The FICI of the antibiotic and phytochemical combinations, as well as the kinetics of bacterial growth inhibition, were graphically represented using Origin Pro 8 software (OriginLab, Northampton, MA, USA).

## 5. Conclusions

The terpenoid compounds CAR and THY effectively inhibit the growth of both MSSA and MRSA strains. In contrast, compounds from the terpene group, such as sabinene, 3-carene, and α-pinene, exhibit limited activity against MRSA, even at higher concentrations. The observed synergistic interactions between CAR and THY when combined with conventional antibiotics highlight their potential to enhance antibacterial efficacy and combat antibiotic resistance. Furthermore, these terpenoids were effective against biofilms formed by MSSA and MRSA, which are critical for their pathogenicity. Results indicate that CAR and THY can disrupt established biofilms, with CAR specifically improving the efficacy of antibiotics, presenting a promising solution for biofilm eradication.

Given the strain-dependent nature of these interactions, it is essential to carefully evaluate specific combinations against individual strains, particularly in MRSA infections, to develop personalized treatment strategies. Future research should focus on elucidating the mechanisms of action, optimizing these combinations for clinical application, and conducting in vivo studies to validate the findings. In conclusion, this study offers significant insights into potential alternative antimicrobial strategies/approaches and underscores the potential of plant-derived compounds as promising agents in addressing the growing challenge of antibiotic-resistant bacterial infections.

## Figures and Tables

**Table 1:** Geometric mean of minimum inhibitory concentration (MIC) of essential-oil-derived active compounds and antibiotics on *S. aureus* reference strains.

			MIC (μg mL^−1^)									
	Chemical Class	Density (g mL^−1^)	MSSA				MRSA					
			ATCC 11632	ATCC 6538	ATCC 25923	ATCC 43300	ATCC 33591	ATCC BAA-1708	ATCC BAA-2312	ATCC 700699	ATCC 700698	NCTC 12493
α-pinene	Terpene	0.0858	≥2048.0	≥2048.0	≥2048.0	>2048.0	>2048.0	>2048.0	>2048.0	>2048.0	>2048.0	2048
(R)-(+)-limonene		0.842	>2048.0	>2048.0	>2048.0	>2048.0	>2048.0	>2048.0	>2048.0	>2048.0	>2048.0	>2048.0
sabinene		0.842	2048.0	2048.0	2048.0	≥2048.0	≥2048.0	>2048.0	>2048.0	>2048.0	>2048.0	>2048.0
3-carene		0.857	1625.5	>2048.0	>2048.0	2048.0	1024.0	>2048.0	>2048.0	>2048.0	>2048.0	>2048.0
α-terpinene		0.837	>2048.0	>2048.0	>2048.0	>2048.0	>2048.0	>2048.0	>2048.0	>2048.0	>2048.0	>2048.0
ℽ-terpinene		0.850	>2048.0	>2048.0	>2048.0	>2048.0	>2048.0	>2048.0	>2048.0	>2048.0	>2048.0	>2048.0
terpinolene		0.861	>2048.0	>2048.0	>2048.0	>2048.0	>2048.0	>2048.0	>2048.0	>2048.0	>2048.0	>2048.0
(−)-terpinene-4-ol	Terpenoid	0.934	1722.2	2048.0	2048.0	>2048.0	>2048.0	>2048.0	>2048.0	>2048.0	>2048.0	>2048.0
thymol		0.965	256.0	512.0	724.1	512.0	512.0	>2048.0	>2048.0	2048	>2048.0	2048
carvacrol		0.976	128.0	203.2	181.0	430.0	362.0	861.1	1024.0	861.1	724.1	724.1
CAZ	Antibiotics ^1^	-	16.0	8.0	32.0	≥64.0	>64.0	>64.0	>64.0	>64.0	>64.0	>64.0
CTX		-	8.0	>64.0	4.0	32.0	>64.0	32.0	32.0	>64.0	>64.0	>64.0
STR		-	8.0	2.0	4.0	4.0	>64.0	16.0	8.0	8.0	8.0	>64.0
CHL		-	16	8.0	16.0	8.0	64.0	16.0	8.0	4.0	8.0	8.0
TET		-	2	<0.5	0.5	1.0	>64.0	1.0	1.0	64.0	64.0	>64.0
AMC		-	1	<0.5	0.5	32.0	>64.0	>64.0	32.0	>64.0	>64.0	>64.0
GEN		-	<0.5	<0.5	<0.5	64.0	8.0	1.0	1.0	>64.0	>64.0	1.0
CIP		-	<0.5	<0.5	<0.5	0.5	0.5	64.0	<0.5	16.0	16.0	1.0
OXC		-	<0.5	<0.5	<0.5	4.0	4.0	2.0	1.0	>64.0	>64.0	32.0
VAN		-	2.0	2.0	2.0	2.0	2.0	1.0	1.0	8.0	2.0	1.0

^1^ CAZ—ceftazidime, CTX—ceftriaxone, STR—streptomycin sulfate, CHL—chloramphenicol, TET—tetracycline, AMC—amoxicillin clavulanic acid, GEN—gentamicin, CIP—ciprofloxacin, OXC—oxacillin, and VAN—vancomycin.

**Table 2:** Quantification of biofilm.

Strains	MHB		MHB + 1% Glucose	
	Incubation Period (h)			
	24	48	24	48
ATCC 11632	nonadherent	nonadherent	nonadherent	nonadherent
ATCC 6538	strongly	strongly	strongly	strongly
ATCC 25923	nonadherent	nonadherent	strongly	strongly
ATCC 43300	nonadherent	nonadherent	weakly	moderately
ATCC 33591	nonadherent	nonadherent	weakly	nonadherent
ATCC BAA−1708	weakly	weakly	strongly	strongly
ATCC BAA−2312	moderately	moderately	strongly	strongly
ATCC 700699	weakly	nonadherent	weakly	moderately
ATCC 700698	weakly	nonadherent	nonadherent	weakly
NCTC 12493	weakly	moderately	strongly	strongly

**Table 3:** Biofilm eradication effect of antibiotics and phytochemicals alone.

Agent	Type of Effect	*S. aureus* Strains							
		MSSA ATCC 6538				MRSA ATCC BAA−2312			
		MIC	2 × MIC	4 × MIC	PEC (µg mL^−1^) ^3^	MIC	2 × MIC	4 × MIC	PEC (µg mL^−1^)
CAZ	I ^1^	28.5	59.8	70.6	>32.0				
	II ^2^	0.0	0.0	0.0					
CHL	I	51.6	56.6	60.3	>32.0	94.0	96.0	97.6	8.0
	II	0.0	0.0	0.0		65.0	53.5	74.3	
STR	I	34.8	39.4	64.9	>8.0	46.8	47.7	61.8	≤8.0
	II	0.0	0.0	0.0		47.0	30.4	0.0	
VAN	I	49.3	50.3	51.0	>16.0	23.2	96.6	98.4	2.0
	II	0.0	0.0	0.0		32.0	72.0	74.3	
CAR	I	56.5	41.6	22.6	256.0	44.2	88.5	89.6	2048.0
	II	89.8	95.4	95.6		32.6	80.3	86.0	
TYM	I	40.3	47.0	48.2	1024.0				
	II	0.0	54.6	65.4					
CTX	I					89.7	90.8	84.5	32.0
	II					82.2	85.5	86.2	
TET	I					92.2	94.7	96.8	1.0
	II					54.0	58.0	51.0	
GEN	I					20.5	21.0	51.4	2.0
	II					32.3	50.5	19.2	
OXC	I					91.0	92.3	96.2	1.0
	II					71.1	70.8	73.3	

^1^ I—Biofilm eradication (%); ^2^ II—Planktonic cell eradication (%); ^3^ PEC—Planktonic cell eradication concentration.

**Table 4:** CAR + STR and CAR + GEN against biofilm formed by ATCC BAA-2312 and CHL + TYM, CHL + CAR, and CAR + TYM against biofilm formed by ATCC 6538.

MIC Combinations	CAR + STR		CAR + GEN		CHL + TYM		CHL + CAR		CAR + TYM	
	BR ^1^ (%)	PCR ^2^ (%)	BR (%)	PCR (%)	BR (%)	PCR (%)	BR (%)	PCR (%)	BR (%)	PCR (%)
4 × MIC	83.5	85.6	0.0	96.1	0.0	92.4	0.0	93.1	0.0	92.4
2 × MIC	87.1	85.3	34.7	96.6	0.0	94.8	47.0	90.2	0.0	94.8
MIC	87.6	76.3	38.3	90.7	52.1	89.3	44.0	89.4	52.1	89.3
1/2 × MIC	75.0	34.6	64.4	85.1	68.1	87.0	83.4	81.6	68.1	87.0
1/4 × MIC	31.0	24.1	78.3	56.2	74.6	44.7	79.3	83.9	74.6	44.7
PEC ^3^ (µg mL^−1^)	1024.0 + 8.0		1024.0 + 1.0		2.0 + 128.0		2.0 + 50.8		101.5 + 256.0	

^1^ BR—Biofilm removal, ^2^ PCR—Planktonic cell removal, ^3^ PEC—Planktonic cell eradication concentration.

## Data Availability

The data that support the findings of this study are available from the corresponding author upon reasonable request.

## Supplementary Materials

The following supporting information can be downloaded at: https://www.mdpi.com/article/10.3390/antibiotics13111030/s1, Table S1. Pre-checkerboard combination of phytochemicals and antibiotics against MSSA and MRSA strains.
