# Supplementary material for: Anti-Staphylococcus aureus Activity of Volatile Phytochemicals and Their Combinations with Conventional Antibiotics Against Methicillin-Susceptible S. aureus (MSSA) and Methicillin-Resistant S. aureus (MRSA) Strains
Source: Antibiotics (Basel). 2024 Oct 31;13(11):1030. doi: 10.3390/antibiotics13111030 (PMC11591321; doi:10.3390/antibiotics13111030)
Supplement: Supplementary file 1 [file antibiotics-13-01030-s001.zip › antibiotics-3263075-supplementary.pdf]

Table S1. Pre-checkerboard combination of phytochemicals and antibiotics against MSSA and MRSA strains

Combination		MSSA					MRSA				
Antibiotics	Phytochemicals	ATCC 25923	ATCC 11632	ATCC 6538	ATCC 43300	ATCC 33591	ATCC BAA-2312	ATCC BAA-1708	ATCC 700699	ATCC 700698	NCTC 12493
CAZ	CAR	- [3]	-	-	-	/ [1]	/	/	/	/	/
CTX		-	-	/	-	/	+	+	/	/	/
STR		-	-	-	+	/	+	+	+	+	/
CHL		-	-	+	-	-	-	-	+	+	+
TET		/	+ [4]	/	-	/	-	-	+	-	/
AMC		/	-	/	-	/	-	/	/	/	/
GEN		/	/	/	+	+	+	+	/	/	+
CIP		/	/	/	-	-	/	-	-	+	+
VAN		-	-	-	-	-	-	-	-	-	+
OXC		/	/	/	-	-	-	-	/	/	+
CAZ	THY	-	-	-	-	/	/	/	/	/	/
CTX		-	-	/	-	/	/	/	/	/	/
STR		-	-	-	+	/	/	/	/	/	/
CHL		-	-	+	-	-	/	/	/	/	/
TET		/	+	/	-	/	/	/	/	/	/
AMC		/	-	/	-	/	/	/	/	/	/
GEN		/	/	/	-	+	/	/	/	/	/

CIP		/	/	/	-	-	/	/	/	/	/
VAN		-	-	-	-	-	/	/	/	/	/
OXC		/	/	/	-	-	/	/	/	/	/
GEN	3-carene	/	/	/	/	+	/	/	/	/	/
CIP		/	/	/	/	-	/	/	/	/	/
CHL		/	/	/	/	-	/	/	/	/	/
VAN		/	/	/	/	-	/	/	/	/	/
CAR	THY	-	-	+/- [2]	-	-	/	/	/	/	/

CAR-carvacrol, TYM-thymol, CAZ-ceftazidime, CTX-ceftriaxone, STR-streptomycin sulfate, CHL-chloramphenicol, TET-tetracycline, AMC-amoxicillin clavulanic acid, GEN-gentamicin, CIP-ciprofloxacin, VAN-vancomycin, and OXC-oxacillin

[1] / No combination was performed for the specified strain

[2] +/- Weak synergy

[3] – No synergistic effect was observed

[4] + Synergism was observed
